# MRS-guided HDR brachytherapy boost to the dominant intraprostatic lesion in high risk localised prostate cancer

**DOI:** 10.1186/1471-2407-10-472

**Published:** 2010-09-01

**Authors:** Aleksandra Kazi, Guy Godwin, John Simpson, Giuseppe Sasso

**Affiliations:** 1 Department of Medical Physics, Townsville Teaching Hospital, Townsville, Queensland, Australia; 2 Department of Medical Physics, Auckland Radiation Oncology, Auckland, New Zealand; 3 Department of Radiation Oncology, "Centre Medical de Forcilles" PSPH Hospital, Ferolles-Attilly, France; 4 Faculty of Medicine, Health and Molecular Sciences, James Cook University, Townsville, Queensland, Australia

## Abstract

**Background:**

It is known that the vast majority of prostate cancers are multifocal. However radical radiotherapy historically treats the whole gland rather than individual cancer foci.

Magnetic resonance spectroscopy (MRS) can be used to non-invasively locate individual cancerous tumours in prostate. Thus an intentionally non-uniform dose distribution treating the dominant intraprostatic lesion to different dose levels than the remaining prostate can be delivered ensuring the maximum achievable tumour control probability.

The aim of this study is to evaluate, using radiobiological means, the feasibility of a MRS-guided high dose rate (HDR) brachytherapy boost to the dominant lesion.

**Methods:**

Computed tomography and MR/MRS were performed for treatment planning of a high risk localised prostate cancer. Both were done without endorectal coil, which distorts shape of prostate during the exams.

Three treatment plans were compared:

- external beam radiation therapy (EBRT) only

- combination of EBRT and HDR brachytherapy

- combination of EBRT and HDR brachytherapy with a synchronous integrated boost to the dominant lesion

The criteria of plan comparison were: the minimum, maximum and average doses to the targets and organs at risk; dose volume histograms; biologically effective doses for organs at risk and tumour control probability for the target volumes consisting of the dominant lesion as detected by MR/MRS and the remaining prostate volume.

**Results:**

Inclusion of MRS information on the location of dominant lesion allows a safe increase of the dose to the dominant lesion while dose to the remaining target can be even substantially decreased keeping the same, high tumour control probability. At the same time an improved urethra sparing was achieved comparing to the treatment plan using a combination of EBRT and uniform HDR brachytherapy.

**Conclusions:**

MRS-guided HDR brachytherapy boost to dominant lesion has the potential to spare the normal tissue, especially urethra, while keeping the tumour control probability high.

## Background

It is known that the vast majority of prostate cancers are multifocal. However radical radiotherapy historically treats the whole gland rather than eradicate individual cancer foci and true optimization of dose distributions in prostate cancer is not performed due to uncertainties in the position of dominant intraprostatic lesion (DIL) within the prostate. Provided the areas of tumour burden can be localized within the prostatic volume, an intentionally non-uniform dose distribution treating the dominant intraprostatic lesion (DIL) and the remaining prostate to a dose ensuring the maximum achievable tumour control probability (TCP) can be delivered.

The magnetic resonance spectroscopy (MRS) can be used non-invasively to diagnose and locate cancerous tumours in the prostate[1]. The majority of MRS investigations of prostate cancer employ proton MRS (1 H MRS) with molecules studied including choline, citrate, lactate and creatine, as well as water and lipids. Areas significantly infiltrated by prostate adenocarcinoma have higher choline to citrate ratio on MRS investigation compared to normal prostatic tissue and benign hypertrophy [1,2].

There are a number of studies supporting the value of MRS for prostate cancer diagnosis and treatment planning. Studies of MRS to direct prostate biopsy show that the use of combination MRI and MRS may reduce the rate of false-negative biopsies and hence decrease the need for more extensive biopsy protocols and/or repeated biopsy procedures [3]. The combination of volumetric data from MRS and anatomical display of MR improves the evaluation of extracapsular extension (ECE) [4]. MRS can diagnose metabolic atrophy which is indicative of successful treatment because the growth of normal or abnormal cells cannot occur without metabolism. Thus MRS also has the potential to be an earlier indicator for resolution of local disease than the PSA nadir [5,6]. The ratio of (choline + creatine)/citrate from MRS examination was also found to correlate with the Gleason grade from biopsy, which in most cases correlates to the aggressiveness of prostate cancer [7].

A number of studies investigated the feasibility of including MRSI data into radiotherapy treatment planning for prostate cancer [8-12]. These studies show that the radiotherapy planning of intensity modulated radiotherapy (IMRT) as well as low dose rate (LDR) brachytherapy to boost the dose to MRS-defined regions is technically feasible with both of these treatment techniques.

The aim of this study is to evaluate the feasibility and the impact of a MRS-guided high dose rate (HDR) brachytherapy boost to the DIL in prostate cancer by comparing the radiobiological outcome of different treatment modalities in a case study of a prostate cancer patient.

## Methods

### Study

A planning study of dose escalation to a MRS-defined dominant lesion with a combination of external beam therapy and the HDR brachytherapy was performed. This study is part of our broader research project investigating the use of MRS in prostate radiotherapy. The project has been granted ethics approval by the Townsville Hospital Ethics Committee for the additional MR/MRS investigation and use of images. Patient informed consent was obtained.

### Patient

A patient with a high risk localised prostate cancer, no history of prior radiotherapy and no evidence of distant metastatic disease, was chosen for this study.

The volume of prostate was 63cm^3^. Six specimens of tissue were obtained with transrectal ultrasound sextant (TRUS) biopsy, which was the standard approach at the Townsville Hospital at the time of this study. Each biopsy specimen consisted of thin fragments of tissue measuring 12-18 mm in length. The biopsy revealed adenocarcinoma of the prostate in the three cores on the right side and none in the three cores on the left side of the prostate, with 100%, 70% and 30% of core involvement in the right superior, mid and inferior cores, respectively. Gleason score was 3+4 = 7 for all the cancer-positive cores. The prostate specific antigen (PSA) at diagnosis was 5.3 ng/ml and the clinical stage from digital rectal examination and the MRI was T3a according to AJCC-TNM[13].

### Imaging

In addition to the conventional computed tomography (CT) for treatment planning, a combined MR/MRS examination was performed. The patient was scanned with bowel and bladder preparation during both CT and MR/MRS acquisitions to improve reproducibility according to the following protocol:

- Bladder and rectal volume should not vary between simulation CT, MRI and treatments

- Bladder should be emptied "twice" one hour prior to scan/treatment, patient should drink two glasses of water soon thereafter

- Fibogel should be started one week before planning and one week before treatment

Consider administering glycerine suppositories 30 min prior to scan and treatment unless bowel movement (less than 1 hour before)

The CT and MRI scans were performed using the same immobilization system applied to radiotherapy planning, including head rest, knee support and feet rest.

MR images were obtained with a 1.5T clinical scanner (Magnetom Symphony, Siemens, Erlangen, Germany), without contrast. To minimise examination time and improve patient's compliance only the following MR sequences were performed: For the morphological study, T2-weighted (T2W) turbo spin echo (TSE) images with high spatial resolution, 3 mm slice thickness with no gap between slices, were acquired in the transversal plane including the entire prostate and seminal vesicles; A three plane T2W study was conducted, including the T2W true fast imaging with steady-state precession (FISP) axial, coronal and sagittal. A belt was placed over the phase array coil to reduce the effect of motion.

The magnetic resonance spectroscopic imaging used the point resolved spatially localized spectroscopic sequence (PRESS) with fat and water spectral suppression and T_E_/T_R _= 135/1500. Magnetic field homogeneity in the volume of interest was ensured by manual shimming the water resonance peak. We have found that when using the 1.5T scanner and no endorectal coil, the manual shimming was crucial to obtaining spectra with acceptable signal-to-noise ratio.

Shimming for two-dimensional (2D) MRSI was found to be easier and faster than for three-dimensional (3D) MRSI, as it was carried out only for the thickness of one slice. A body coil positioned directly above the prostate and one of the spine coils directly below prostate volume, were used instead of an endorectal (ER) coil for the MR signal reception. The total MR/MRS session was approximately 45 minutes long.

Positioning of the MRS volume of interest (VOI) in the superior-inferior direction was guided by the underlying 3D axial T2 morphological images, in order to include the level suspected to contain the dominant intraprostatic lesion. The VOI was adjusted to completely cover the prostate on the selected transverse slice, with an additional row of voxels around the prostate. The voxel size was 10x10x10 mm^3^. With further optimisation of our MRS technique we were able to decrease the voxel size to 7x7x7mm^3 ^while keeping a similar signal to noise ratio. However all patients scanned using the smaller voxel size had multiple dominant lesions in the prostate, thus could not be used for this study as the dose escalation to the dominant lesions would not be practical.

Positioning of the 2D MRS slice in the inferior-posterior and lateral directions was critical to obtain a minimum overlap of different tissue types within each voxel.

The dominant intraprostatic lesion (DIL) was located on the co registered MR/MRS as the region of hypointensity and was confirmed by MRS to be an aggressive cancer. The DIL was subsequently delineated according to this region of hypointensity on MRI, as MRS didn't have sufficient resolution for accurate delineation, even if the smaller voxel size of 7x7x7mm^3 ^was used. The resolution of MRS could be improved if the voxel size was decreased; however it would also mean decreasing the MRS signal strength. Further progress in signal acquisition and technology is necessary to improve the signal-to-noise ratio, thus allowing a smaller MRS voxel size.

### Treatment planning

CT and MR images were transferred to the CMS XiO treatment planning system for external beam treatment planning. MR images were also transferred to the Nucletron Plato treatment planning system for high dose rate (HDR) brachytherapy planning, as the DIL could be precisely localized on the MR images. Normal structures delineated on CT images were left and right femoral head and necks, rectum and patient outline, normal structures delineated on the MRI: urethra and rectum. The definitions of clinical and planning target volumes are summarized in Table 1.

**Table 1:** Target volumes definition.

CTV_CT _= prostate gland + base of seminal vesicles, delineated on CT
CTV_MR _= prostate gland only, delineated on MRI
CTV_DIL _= dominant intraprostatic nodule, delineated on MRI/MRSI
PTV_EBRT _= CTV_CT _+ 10 mm margin in all directions but posteriorly where 5 mm where used
PTV_HDR _= CTV_MR_
PTV_DIL _= CTV_DIL_

"CTV" is the clinical target volume, "PTV" - the planning target volume, subscript "EBRT" refers to the external beam radiotherapy, "HDR" - to the high dose rate brachytherapy, "DIL" - the dominant intraprostatic lesion.

Three treatment plans were prepared:

Plan A

- external beam radiation therapy (EBRT) combined with androgen deprivation therapy (ADT) starting 6months prior to the start of EBRT and continuing for two years after completion of EBRT

- conformal plan utilizing five 10MV photon beams at gantry angles of 0°, 54°, 83°, 253° and 282°

- prescription dose: 74 Gy delivered in 37 fractions

- for the PTV_EBRT_: V95 to be equal 100%; however in this case, due to rectum DVH being close to tolerance and the particular rectal filling, the radiation oncologist accepted the plan with V95 of 98% for the PTV_EBRT_

Plan B

- combination of EBRT and HDR brachytherapy

- EBRT plan delivering 60Gy to PTV_EBRT _in 30fractions

- HDR brachytherapy delivering 10Gy to the PTV_HDR _in one fraction

Plan C

- combination of EBRT and HDR brachytherapy with a synchronous integrated boost (SIB)

- EBRT plan delivering 60Gy to PTV_EBRT _in 30fractions to prostate

- HDR brachytherapy delivering one dose level to the PTV_HDR _and a boost to the PTV_DIL _in one fraction, where dose levels are optimized to maximise the TCP and at the same time, minimise the doses to the critical normal organs; these final optimized dose levels were 7.5Gy for the PTV_HDR _and a concomitant boost of 7.5Gy to the PTV_DIL_

The plan 'A' dose constrains for organs at risk were as follows:

- rectum: less then 4% of volume should receive more then 74Gy; less than 20% - 70Gy; less than 30% - 60Gy and less than 70% - 30Gy.

- femoral head and necks: less then 60% of volume should receive more then 40Gy and less then 40% of volume - more then 60Gy.

These dose constrains were derived from the results of a consensus process on the Australia and New Zealand radiotherapy standards, initiated by the Faculty of Radiation Oncology Genito-Urinary Group[14]. Dose of more than 70Gy was prescribed to at least 99% of PTV_EBRT_. For the EBRT part of plans 'B' and 'C' only 30 fractions of this plan were planned to be delivered.

During HDR brachytherapy planning, care was taken for the 100% prescription dose to fully encompass the PTV_HDR_, and additionally for the second brachytherapy plan, for the 15Gy isodose to fully encompass the PTV_DIL_. A maximum of 15Gy delivered to the urethra was accepted and the dose to the rectum was minimised as long the target coverage was not compromised.

### Treatment plan evaluation

The plans were compared in terms of minimum, maximum and average doses to the targets and organs at risk, the dose volume histograms as well as biologically effective doses for organs at risk and tumour control probability (TCP) for the target volumes.

Radiobiological modelling was used to compute and compare the TCP for a range of tumour sensitivity for all three plans. Calculation of TCP was based on the linear-quadratic (LQ) model including repopulation according to the Equation 1, where *SF *is the surviving fraction, *D *- the total dose, and *d *- dose per fraction, α- the initial slope of a cell survival curve, β- the curvature of the cell survival curve, *t*_*treatment *_- time of the treatment, *T*_*repop *_- doubling time (equal to 42days).

(1) $$SF=\exp\left[-\alpha D-\beta nd^{2}-t_{treatment}\cdot \frac{\ln2}{T_{repop}}\right]$$

For combined treatment modalities of external therapy and brachytherapy, the combined clonogen survival *S *can be calculated according to the Equation 2:

(2) $$S=S_{EBRT}\cdot S_{HDR}$$

TCP was calculated according to the Equation 3, where *N*_*0 *_is the initial number of cancer clonogens.

(3) $$TCP=exp(-N_{0}S)$$

There was much discussion following the clinical evidence supporting the idea that the prostate tumours have exceptionally low α/β values [15]. Therefore we have employed two sets of radiobiological data for the prostate cancer. One dataset assumes the α/β =1.5Gy, α =0.0391Gy^-1 ^and the number of clonogens *N*_0 _=290[16]. A second dataset assumes the α/β =3.1Gy, α =0.15Gy^-1 ^and *N*_0 _=10^6^-10^7 ^[17]. These two α/β ratios, as pointed out by Fowler [18], differ in the assumption of the time of onset of the repopulation, which is late for the α/β =1.5Gy and early (of 0 or 28 days after starting the treatment) for the α/β =3.1Gy. Thus here the repopulation for the α/β =1.5Gy dataset was not taken into account, and was assumed to start at the beginning of treatment for the α/β =3.1Gy dataset. Additionally, a ratio of clonogens in the DIL to rest of prostate was assumed to be 90:10 [19].

For combination of modalities and different fractionation schemes, the dose has to be recalculated to the BED dose, corresponding to a 2Gy radiation fraction. The linear quadratic model can be used to calculate the biologically effective dose (*BED*) according to Equation 4. The α/β ratio for normal tissues was assumed to be 3.

(4) $$BED=D\left(1+\frac{d}{\alpha /\beta }\right)$$

## Results

### MRSI

**Figure 1:**
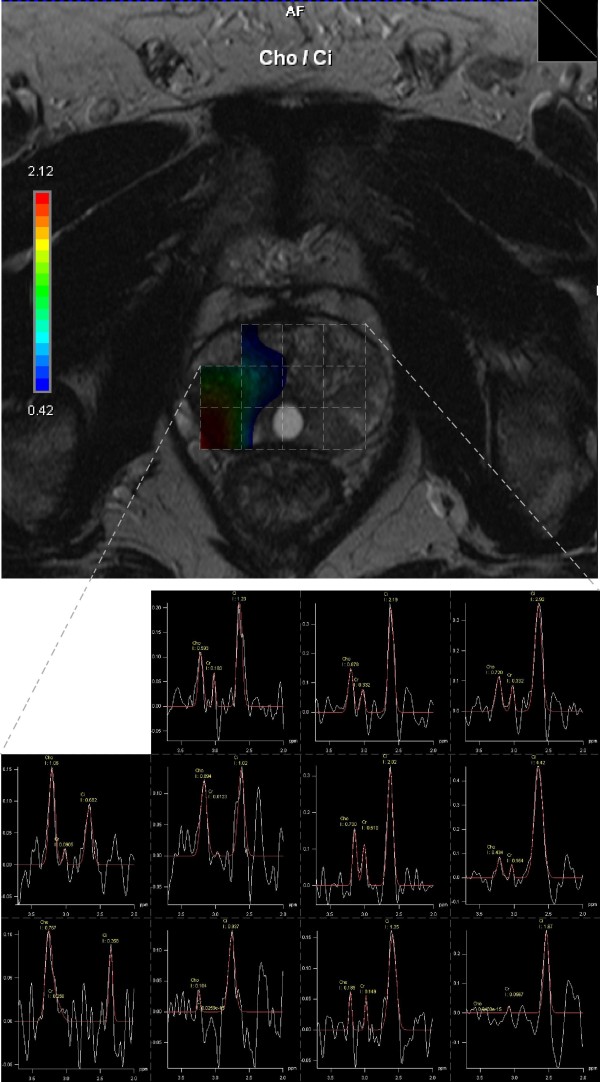
**Up: T2-weighted transverse MR image, with the MRSI grid and a color map corresponding to the choline/citrate ratio**. Down: MRS spectra for respective voxels.

The MRSI results are show on figure 1. On the upper picture all single voxels included in the volume selected in the spectroscopic sequences are graphically represented in a two-dimensional (2D) image as a grid overlying the corresponding T2-weighted transverse image, along with a color map corresponding to the choline/citrate ratio. On the lower picture, the MRS spectra for respective voxels are presented. Due to the thickness of the MRS slice (10mm) and MR axial slice (3mm), the MRS slice covered three MR slices. Therefore, the middle one of the three slices was selected for overlaying the MRS grid for evaluation. Voxels on the right side of prostate, in the region of suspected dominant lesion, show elevated levels of choline, the marker of active tumour growth.

**Figure 2:**
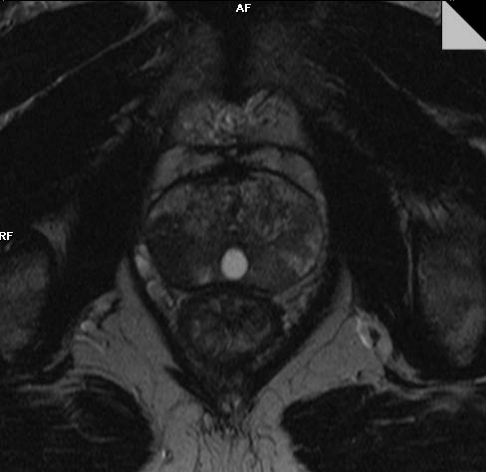
**Underlying MR image to the MRS spectra presented on figure 1A**.

Figure 2 shows the underlying MRI image for comparison.

### External beam plans

**Figure 3:**
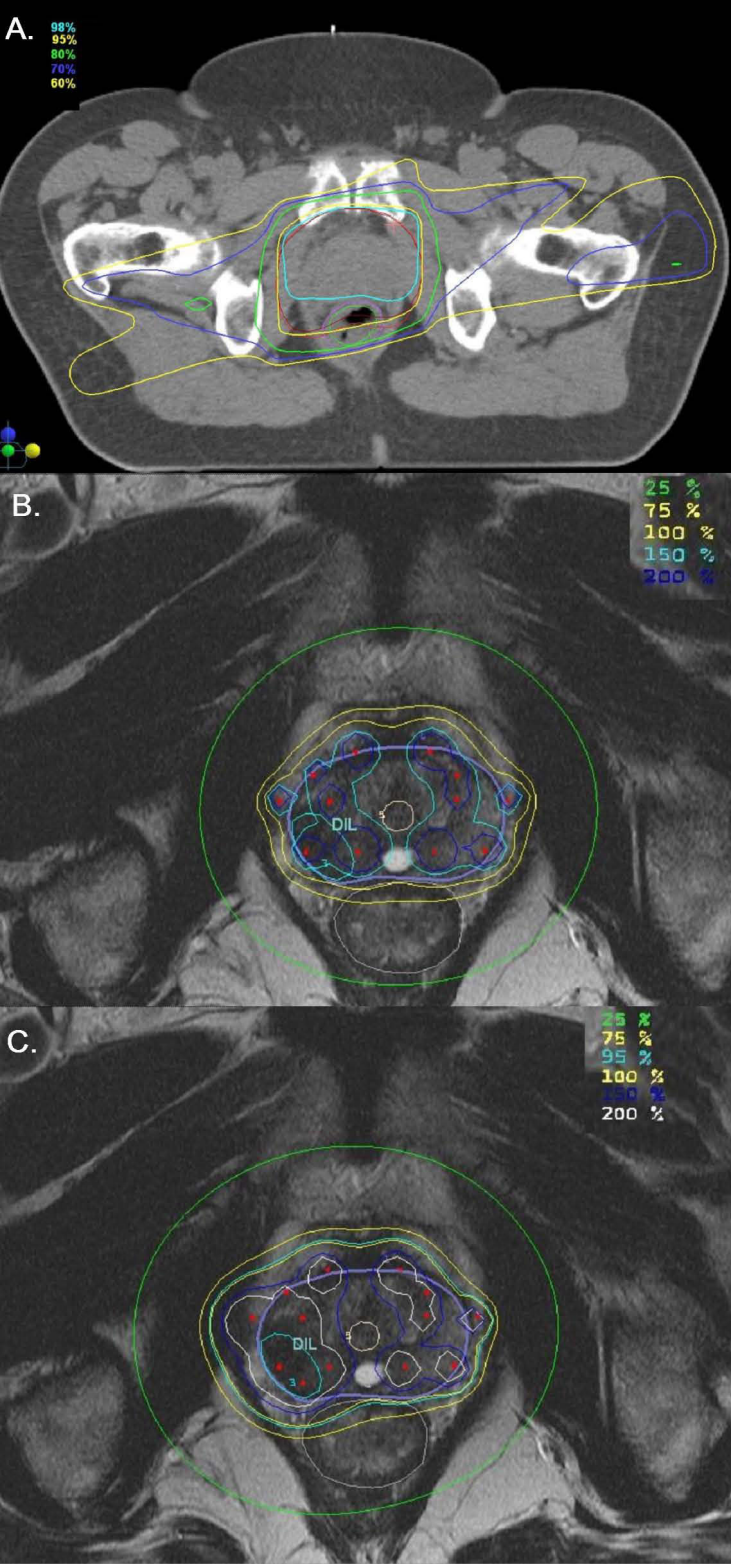
**Dose distributions for the following plans: conformal external beam plans where dose is relative to the prescription dose to the target (A); the HDR brachytherapy plan where the 100% isodose corresponds to 10Gy (B) and the SIB HDR brachytherapy plan where the 100% isodose corresponds to 7.5Gy (C)**.

**Figure 4:**
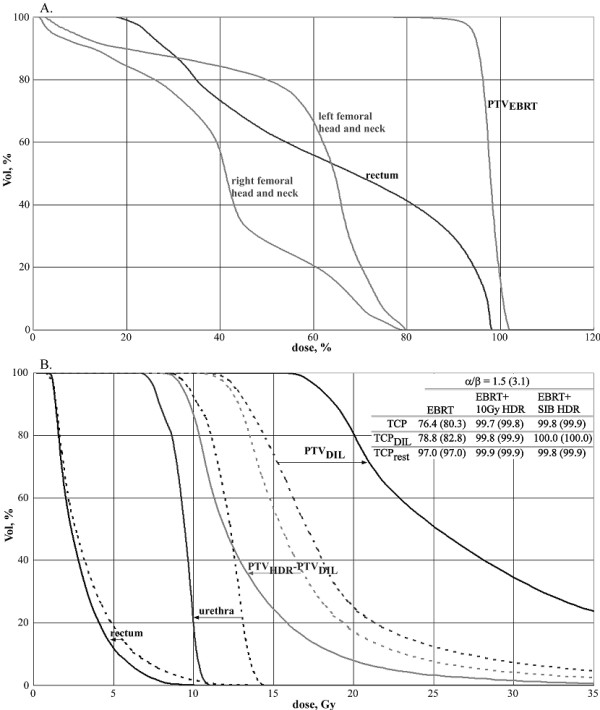
**Dose volume histograms (DVHs) for the conformal external beam plans where dose is relative to the prescription dose to the target (A), and for the HDR brachytherapy plans (B) delivering 10Gy to the whole prostate (dashed lines) and the SIB HDR brachytherapy plan (continuous lines)**. Table on Figure B. shows TCP values for the investigated treatment plans.

Figure 3A shows the dose distribution and figure 4A - the dose volume histograms (DVHs) for the conformal external beam plans where the dose is relative to the PTV_EBRT _prescription dose. DVHs shown are for PTV_EBRT_, rectum, right femoral head and neck and left femoral head and neck. The PTV_EBRT _volume wraps around the rectum on the right side, making it a very difficult treatment case for the external conformal beam therapy planning. The doses to the femoral head and necks as well as the rectum were pushed towards the tolerances of these organs to achieve good coverage of PTV_EBRT _and for a realistic comparison of TCP values between the different radiotherapy delivery methods. The results are shown in table 2.

**Table 2:** Dose-volume parameters for the PTV_EBRT _and CTV_CT _for the 74Gy external beam plan.

	**PTV**_**EBRT**_	**CTV**_**CT**_
V90	1.00	1.00
V95	0.98	1.00
V100	0.18	0.32
V125	0.00	0.00

D100, Gy	69.60	72.00
D90, Gy	71.70	73.00

D_min_, Gy	67.42	71.06
D_max_, Gy	74.43	74.45
D_av_, Gy	73.04	73.69

V90, V95, V100 and V125 are volumes irradiated to 90, 95, 100 and 125% of prescription dose of 74Gy, respectively. D100 and D90 are doses to the 100% and 90% of volume, respectively.

The resulting TCP was 76.4% with TCP of 78.8% for the DIL and 97.0% for the rest of PTV_EBRT_, calculated for the α/β ratio of 1.5. The TCP vales for both for α/β ratio of 1.5 and 3.1 are presented on Figure 4.

### HDR brachytherapy plans

The HDR brachytherapy plan delivering 10Gy to whole PTV_HDR _resulted in TCP values of 99.7% and 99.8% for α/β ratio of 1.5 and 3.1, respectively.

In the SIB HDR brachytherapy plan dose levels were optimized to match the TCP of the 10Gy HDR brachytherapy plan for the PTV_DIL _and remaining PTV_HDR_. As for the standard brachytherapy plan the TCP was close to 100%, it was found that to achieve the same level of tumour control, doses of just 7.5Gy to PTV_HDR _and an additional 7.5Gy boost to the PTV_DIL _were needed.

Figures 3B and 3C show the dose distribution for the HDR brachytherapy plans delivering 10Gy to the PTV_HDR _and the SIB HDR plan, respectively.

Figure 4B shows the dose volume histograms for the HDR brachytherapy plans delivering 10Gy to the PTV_HDR _(dashed lines) and the SIB HDR plan delivering 7.5Gy to the PTV_HDR _and a concomitant boost of 7.5Gy to the PTV_DIL _(continuous lines). The DVHs for the PTV_DIL_, PTV_HDR _excluding PTV_DIL_, rectum and urethra are shown. The HDR brachytherapy plan delivering 10Gy to the PTV_HDR _used 12 needles, while the SIB HDR brachytherapy plan used 14 needles.

The minimum, maximum and average doses to rectum, bladder, PTV_HDR _excluding PTV_DIL_, PTV_DIL _and urethra; as well as volume irradiated to 90, 100, 125, 150 and 200% of prescription dose (V90, V100, V125, V150 and V200, respectively), dose to the 100% and 90% of volume (D100 and D90, respectively) for PTV_HDR _excluding PTV_DIL_, PTV_DIL _and urethra are shown in table 3. The prescription dose for PTV_HDR_, PTV_DIL _and urethra for 10Gy HDR brachytherapy plan was considered to be 10Gy; for the SIB HDR brachytherapy plan the prescription dose to PTV_HDR _excluding PTV_DIL _and urethra was 7.5Gy and 15Gy for the PTV_DIL_.

**Table 3:** Dose parameters for investigated HDR brachytherapy plans.

	**PTV**_**HDR **_**- PTV**_**DIL**_		**PTV**_**DIL**_		urethra	
	
	10Gy HDR brachytherapy	SIB HDR brachytherapy	10Gy HDR brachytherapy	SIB HDR brachytherapy	10Gy HDR brachytherapy	SIB HDR brachytherapy
V90	1.00	1.00	1.00	1.00	0.98	0.99
V100	1.00	1.00	1.00	1.00	0.90	0.90
V125	0.85	0.86	0.94	0.88	0.34	0.35
V150	0.50	0.54	0.71	0.60	0.00	0.00
V200	0.17	0.27	0.24	0.34	0.00	0.00

D100, Gy	9.75	7.50	11.00	15.75	8.75	6.56
D90, Gy	12.00	9.00	13.00	18.38	10.00	7.50

D_min_, Gy	9.37	7.13	10.77	15.08	8.41	6.36
D_max_, Gy	363.09	240.99	83.20	124.70	14.32	10.74
D_av_, Gy	17.92	14.90	18.84	29.76	11.57	8.72

Table 4 shows the minimum, maximum and average biologically equivalent doses (BED) to organs at risk for all investigated treatment plans. As can be concluded from table 4 and shown on figure 4, the two urethra DVH curves for the two HDR brachytherapy plans are well separated. The SIB HDR brachytherapy plan allowed a substantial shift in the urethra DVH towards lower doses, potentially improving urethra sparing.

**Table 4:** The minimum, maximum and average biologically equivalent doses (BED) to organs at risk for investigated treatment plans.

				HDR brachytherapy			
				
	EBRT, 74Gy			rectum		urethra	
	
	rectum	RtFemH&N	LtFemH&N	10Gy HDR brachytherapy	SIB HDR brachytherapy	10Gy HDR brachytherapy	SIB HDR brachytherapy
BED_min_, Gy	18.09	0.11	1.43	0.97	0.83	31.99	24.34

BED_max_, Gy	123.92	82.99	75.80	105.34	62.24	82.67	62.00

BED_av_, Gy	77.97	19.55	36.81	6.33	5.37	58.30	42.8

## Discussion

Brachytherapy is one of the techniques capable of delivering intentionally inhomogeneous dose distributions to the target volume. Thus it is possible to adopt an approach presented here, where a higher dose, prescribed according to the required tumour control probability derived from radiobiological models, is delivered to the tumour burden and a homogenous dose to the whole prostate while the dose to surrounding normal organs can be maintained or even minimised. A number of studies investigated the feasibility of including MRS data into brachytherapy plans with prostate implants [8,9,20]. These studies found that it is theoretically possible to achieve tumour dose escalation in MRS-identified intraprostatic tumour deposits without concomitant delivery of escalated doses to the urethra.

To our knowledge this is the first study comparing radiotherapy plans for conventional EBRT, combination of EBRT and HDR brachytherapy and MRS-guided SIB. We have investigated the potential impact of adding the MRS data to the HDR brachytherapy planning, where the dose prescription can be further customised to be more patient-specific.

Our study is also unique as both CT/MR were performed without ER coil, which distorts the shape of prostate during the MR/MRS investigation. Thus this study doesn't require image transformation. Although the majority of prostate MRS to date is performed using ER coil, the use of external surface coils for both 2D and 3D MRS of prostate cancer was found to be feasible [21]. Our experience at the Townsville Cancer Centre also confirms that prostate MRSI with a combination of multiple external coils is feasible with diagnostic signal-to-noise ratios.

We have shown that the dose to the DIL can be safely increased while the dose to the remaining PTV can be even substantially decreased keeping the same, high tumour control probability and decreasing the doses to critical normal organs, when compared to the uniform HDR brachytherapy plan. Normal tissue sparing is especially important in case of HDR brachytherapy, where the fraction size is larger then for the EBRT and therefore the BED, which is connected to the clinical effect, is larger than the same dose delivered in standard EBRT fractionation scheme.

As the total radiation dose is not a reliable measure of biological effect when dose per fraction or dose rate is changed, the concept of biologically effective dose (BED) was used to compare doses to normal tissues. BED can be calculated for any dose per fraction if a value of α/β for the appropriate tissue is assumed. The α/β ratio determines the sensitivity of a particular cell type to alterations in radiation fraction size [22]. The effects of BED on freedom from PSA failure at 10years and post-treatment biopsy demonstrate a strong dose-response relationship [23].

In HDR brachytherapy the dose delivered to urethra is one of the main concerns. The risk of urethral stricture treatment after prostate cancer therapy is considerable at 1.1 to 8.4%, being highest after brachytherapy plus external beam radiotherapy or radical prostatectomy, with a delayed onset after radiation treatment [24]. Inclusion of MRS information on the location of the DIL allowed us to develop a radiotherapy plan with an improved urethra sparing while keeping the tumour control probability high. In other patients the degree of normal tissue sparing can vary, depending on the number and size of the dominant lesions as well as their location, mainly the proximity to the urethra and rectum.

T3a patient is not the ideal candidate for focal therapy [25]. However in this case we were able to reliably establish the location of the lesion within prostate and the prostatic volume outside of the DIL still received dose of 60Gy of the EBRT and 7.5Gy of the HDR brachytherapy for the SIB plan.

In a certain group of patients, where multiple DILs are present, the delivery of a boost dose to the dominant lesions can be impractical. Performing MRS was important to establish that a second potentially important lesion does not exist as it could preclude delivery of the boost dose. Thus MRS was not only confirming the results of biopsy and MRI. Another approach would be to increase the number of biopsy samples, however MRS has the advantage of being an non-invasive procedure.

Also due to some of the technical limitations of MRS the advantages of selective boost to the DIL in prostate cancer may not be fully utilised. Although the resolution of MRS is constantly improving, it is still inferior to the resolution of such imaging techniques as MRI. Due to the MRS voxel size very small lesions may be missed. In our study MRSI was not used to fully analyse the size and shape of the DIL, but to confirm the location of the tumour burden, therefore the voxel size was not crucial.

The success of treatment employing the selective boost to the dominant lesion could be monitored with regular PSA tests. Follow-up MRS to detect any remaining choline levels could also be performed; however we are not aware of studies investigating the optimal timing of prostate MRS for radiotherapy follow-up. Repeated MRS investigations could be done, however such an approach would not be cost-efficient. Another option would be performing MRS when indicated by increasing PSA. The saturation biopsy could also be employed in this case.

However in this case even if a recurrence of tumour in another location within the prostate was detected, retreatment would be difficult because of the doses already delivered outside of the DIL.

## Conclusions

MRS-guided HDR brachytherapy boost to DIL has the potential to spare the normal tissue, especially urethra, while keeping the tumour control probability high.

## Competing interests

The authors declare that they have no competing interests.

## Authors' contributions

GS, JS and AK designed the study and optimized the MRS technique. The treatment planning was performed by GS, GG and AK. All authors contributed to the analysis and interpretation of the data.

AK drafted the manuscript; all authors revised it critically and approved the final version.

## Pre-publication history

The pre-publication history for this paper can be accessed here:

http://www.biomedcentral.com/1471-2407/10/472/prepub
